# Development and validation of a mouse model to investigate post surgical pain after laparotomy

**DOI:** 10.1016/j.sopen.2024.06.002

**Published:** 2024-06-18

**Authors:** Juan Martinez, Thomas Maisey, Nicola Ingram, Nikil Kapur, Paul A. Beales, David G. Jayne

**Affiliations:** a School of Chemistry, University of Leeds, Leeds, West Yorkshire LS2 9JT, UK; b Leeds Institute of Medical Research, University of Leeds, Leeds, West Yorkshire LS9 7TF, UK; c School of Mechanical Engineering, University of Leeds, Leeds, West Yorkshire LS2 9JT, UK; d The John Goligher Colorectal Surgery Unit, St. James's University Hospital, Leeds Teaching Hospital Trust, Beckett Street, Leeds, West Yorkshire LS9 7TF, UK

**Keywords:** Pain, Mouse model, Peritoneal trauma, Adhesion, Cytokines, Post operative recovery

## Abstract

**Background:**

Postoperative pain following abdominal surgery is a significant obstacle to patient recovery, often necessitating high analgesic doses associated with adverse effects like cognitive impairment and cardiorespiratory depression. Reliable animal models are crucial for understanding the pathophysiology of post surgical pain and developing more effective pain-relieving strategies.

**Methods:**

We developed a mouse model to replicate peritoneal trauma induced by abdominal surgery. 30 C57BL/6 mice underwent laparotomy, with half undergoing standardised peritoneal abrasion and the rest serving as controls. Mouse recovery was assessed using two validated scoring systems of surgical recovery: Post surgery Severity Assessment (PSSA) and Mouse Grimace Score (MGS). Blood samples were taken for cytokine analysis. Adhesions were evaluated on day 6, and peritoneal tissue was examined for healing markers.

**Results:**

After laparotomy, all mice exhibited expected pain profiles. Mice with peritoneal abrasion had significantly higher PSSA (7.2 ± 1.2 vs 4.68 ± 0.82, *p* ≤ 0.001) and MGS scores (3.62 ± 0.74 vs 0.82 ± 0.40, *p* ≤ 0.05) with slower recovery. Serum inflammatory cytokine levels were significantly elevated in the abraded group, and adhesion formation was higher in this group. Immunohistochemical analysis showed significantly increased expression of α-SMA, CD31, CD68, and F4/80 in peritoneal tissue in the abraded group.

**Discussion:**

A mouse model involving laparotomy and standardised peritoneal abrasion replicates the expected pathophysiological changes following abdominal surgery. It will be a useful model for better understanding the mechanisms of post surgical pain and developing improved pain-relief strategies. It also has utility for the study of intra-abdominal adhesion formation.

**Key message:**

To understand the intricate relationship between peritoneal trauma-induced pain, cytokine response, and post-operative adhesion formation in mouse models for advancing therapeutic interventions and enhancing post-operative recovery outcomes.

## Introduction

Around 300 million surgical operations are performed globally each year [1]. Postoperative pain is common to all surgical procedures and is a major limitation to safe patient recovery. Opioid analgesics are frequently used for pain relief following abdominal surgery but can cause side-effects, including cognitive impairment, cardiorespiratory depression, and gastrointestinal dysfunction. There is a need to better understand the pathophysiology of postoperative pain and to develop better strategies for postoperative pain mitigation.

Abdominal pain involves a convergence of physiological, neurological, and psychological factors that intertwine to create a complex sensory perception [2]. This intricate interplay poses a substantial challenge for developing new pain-relieving strategies [3,4]. Conducting pain studies in humans presents inherent difficulties due to subjectivity, ethical constraints, and practical limitations [5,6]. As a result, animal models, primarily utilising species such as mice and rats, have become extensively employed to explore pain phenomena [7]. However, using animal models introduces other challenges, including accurately quantifying behavioural responses that are analogous to human pain experiences [8].

Abdominal pain exhibits substantial variation among individuals due to genetics, sensitivities, and psychological factors [9,10]. Previous research has shown that abrasion models might more closely replicate the changes seen following abdominal surgery, potentially enabling more accurate predictions of therapeutic outcomes and paving the way for developing novel treatment strategies [11].

In this study, we describe a novel peritoneal abrasion model for investigating pain following abdominal surgery and characterise it using validated scoring systems of surgical recovery, the inflammatory cytokine response, and histological assessment of peritoneal healing. We hypothesize that the peritoneal abrasion model will induce measurable postoperative pain that correlates with specific inflammatory cytokine profiles and histological markers of peritoneal healing. This will pave the way for deeper insights into pain mechanisms, personalised treatments, and improved patient care.

## Materials and methods

### Animals and ethics statement

All experiments were performed according to the UK Animals (Scientific Procedures) Act (1986), The National Centre for the Replacement, Refinement and Reduction of Animals in Research (NC3Rs) Guidance and reported using International Animal Research: Reporting *In Vivo* Experiments (ARRIVE 2.0) Guidelines. Male and female C57BL-6 were purchased from Charles River Laboratory, UK. Animals were 8-weeks old with a mean weight of 24.1 ± 3.9 g. All mice were housed individually in a controlled environment (12 h/12 h light/dark cycle; temperature: 22 ± 2 °C; relative humidity: 30–70 %) for seven days prior to surgery. Experiments were performed under UK Home Office Project Licence PP6019116.

### Experimental setup

30 mice were randomly assigned (1:1) to the abraded and control groups. The mice were anaesthetised using inhalational isoflurane (2 L/min, Isoflurane 2 % (Henry Schein)) and administered 0.1 mg/kg of Vetergesic (AnimalCare Limited, UK) subcutaneously. The abdomen was shaved, and the animal placed on a warm plate at 37 °C for the duration of the procedure. The skin was prepared using Contec Prochlor (Contec). A midline 1 cm incision was made in the lower abdomen and 8/0 vicryl sutures used to elevate the right abdominal wall; the left abdominal wall was left untouched. A standardised 1 × 1 cm abrasion was made to the peritoneum lining the right abdominal wall using a 100-grit sterile Emery Cloth (Black and Decker, US) until the peritoneum was erythematous. Control mice underwent laparotomy alone, without abrasion. The abdominal wall was closed using continuous 8/0 vicryl suture, with 2 clips for skin apposition. Mice were recovered in a warming chamber (mini-thermacage, Datesand, UK) until they regained consciousness and then returned to their initial individual cages. The mice were given an electrolyte replenisher gel (Electro-gel, Bio-serv, New Jersey, USA), and standard mice food.

### Assessing surgery severity and pain levels

**Appendix Fig. A1:**
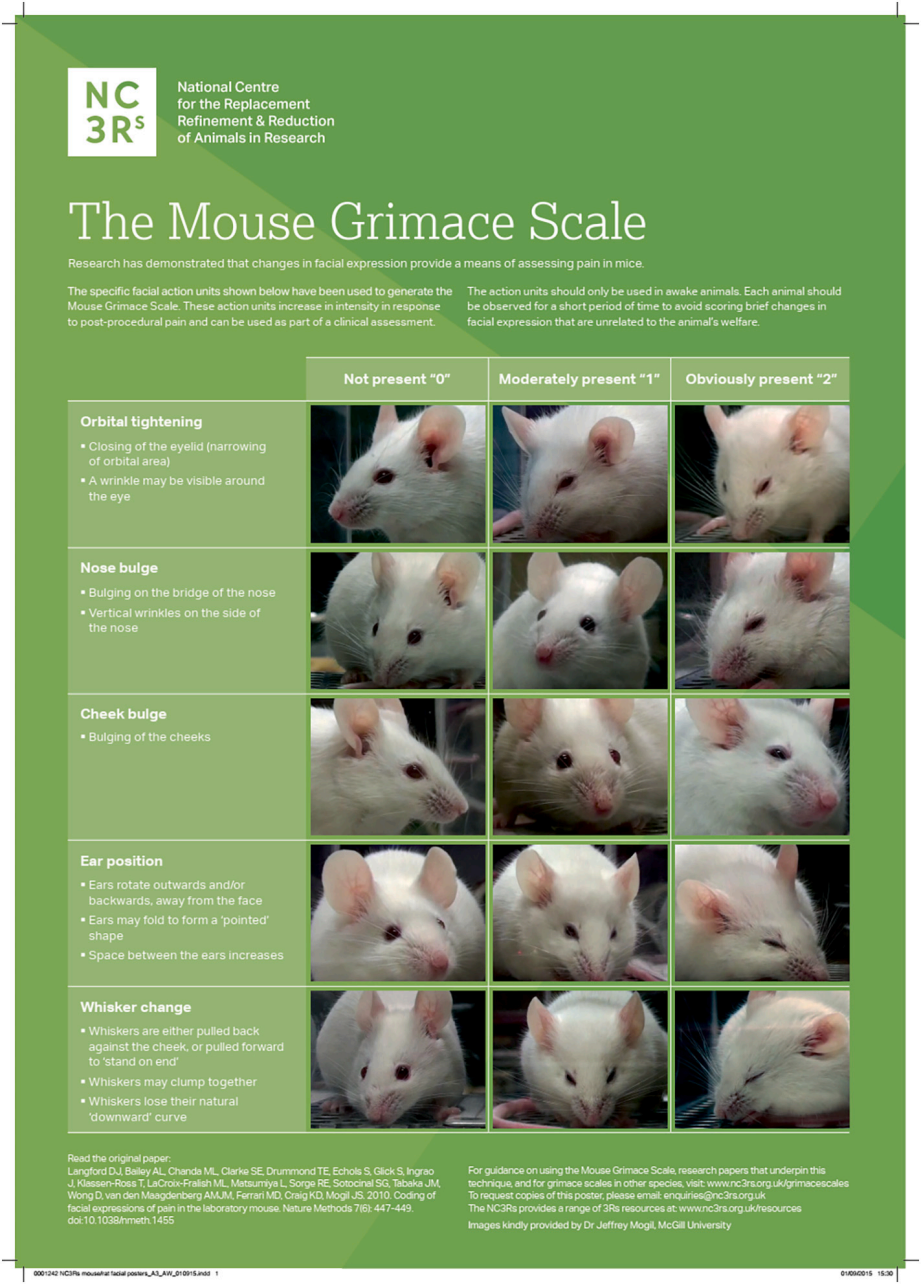
Mouse Grimace Scale provided by the National Centre for the Replacement, Refinement and Reduction of Animals in Research [14].

Two validated scoring systems were used to monitor postoperative recovery. One observer blinded to the treatment groups carried out the mice observations and provided oral analgesia to the mice at the beginning of each day after the surgery in a Vetergesic/Nutella mix as per Pommegaard et al. [12]. The Post surgery Severity Assessment [13] has criteria that include body weight, general condition, behaviour, readiness to walk, and surgery-associated parameters (Appendix Table A1). Each criterion was scored on a scale of 1–20, with detailed information about the state of the mouse at each time-point. At each time point, the individual scores were summated, and action taken if appropriate. A humane endpoint was set at a total score of >20 points, which was deemed to represent excessive suffering, and animals were subjected to Schedule 1 killing. The Mouse Grimace Scale (MGS), developed by the NC3R [14] was used to assess discomfort of the mice after the surgery based on facial expression (Appendix Fig. A1). The MGS considers the orbital tightening, nose bulge, cheek bulge, ear position and whisker change with a scale of 0 (not present), 1 (moderately present), and 2 (obviously present).

Mice were monitored across five timepoints on postoperative day 1 day (2, 4, 6, 8, and 16 h after surgery), and at three timepoints from postoperative days 2 to 6 (9 am, 12.30 pm, and 4 pm).

### Assessing peritoneal adhesions

On postoperative day 6, the animals were humanly euthanised by cervical dislocation. An autopsy was performed through the previous laparotomy wound. The abdomen was inspected for adhesions, which were quantified using the scoring system described by Ito, Shintani [15]. Briefly, adhesions were scored according to their tenacity and morphology on a scale from 0 to 4. Grade 0: No adhesions or insignificant adhesions, Grade 1: Adhesions that are filmy and easy to separate by blunt dissection, Grade 2: Adhesions where blunt dissection is possible but some sharp dissection necessary, Grade 3: Lysis of adhesions possible by sharp dissection only, Grade 4: Lysis of adhesions possible by sharp dissection only, organs strongly attached with severe adhesions. Abraded and unabraded peritoneum was harvested and fixated in 4 % paraformaldehyde for immunohistochemical analysis.

### Serum sampling

On postoperative days 1, 4 and 6, 200 μL of blood was collected from the tail vein of the mice for serum cytokine analysis. Briefly, the tail of the mice was rubbed with EMLA cream 30 min before the procedure and the animal placed in a warming chamber for 10 min. Animals were placed in a restraining device and the lateral tail vein was pierced using a 23G needle. Blood was collected using a micropipette and placed in Eppendorf tubes and left at room temperature for 30 min. Serum was collected by centrifugation (2000 ×*g* for 10 min at 4 °C) and stored at −20 °C.

### Cytokine quantification

Cytokine quantification from serum samples was analysed using a Bio-Plex Pro Mouse Cytokine 7-plex Assay (Bio-Rad, California, USA) according to the manufacturer's instructions. The assay included quantification of IL-1β, IL-2, IL-6, IL-10, IL-12 and TNF-α cytokines. Briefly, a solution of magnetic beads, pre-coated with the detecting antibodies, was added to a 96-well plate and washed using a magnetic handheld device. 50 μL of sample (4× dilution) and calibration curve standards were added into each well and incubated on a shaker at 850 ± 50 RPM at room temperature. The plate was washed using the magnetic handheld device, and 25 μL of detection antibody was added to each well. The incubation step was repeated, and finally 50 μL of Streptavidin, R-Phycoerythrin Conjugate (SAPE) 1× was added. The plate was read using a Bio-Plex MAGPIX Multiplex reader. Data were exported in XLS format (Excel) and analysed using OriginPro 8 (OriginLab, Massachusetts, USA).

### Immunohistochemistry (IHC)

After fixation with 4 % paraformaldehyde, the tissue was processed and embedded in paraffin wax. The peritoneal tissue was placed in a perpendicular orientation to display both the inner and outer layers. Sample blocks were sectioned using a HistoCore BIOCUT manual rotary microtome (Leica Biosystems, Wetzlar, Germany) at 5 μm thickness, mounted onto microscope slides (Fisherbrand Superfrost Plus, Pittsburgh, USA) and placed in an oven at 62 ± 2 °C overnight. The sections were dewaxed with xylene followed by gradual rehydration using decreasing alcohol concentrations. After rehydration, all sections underwent microwave heat-induced epitope retrieval using the retrieval buffer described in Table 1. For 10 min in a domestic microwave at full power with 20 min cooling. Endogenous peroxidases were blocked as described in Table 1, followed by washing with TBS tween (2 × 5 min washes) and 1 × 5 min TBS wash. Antibodies were incubated on the section as described in Table 1 and after washing as previously, HRP-conjugated goat anti-rabbit IgG was incubated with the sections at 1:500 dilution for 30 min at room temperature. DAB was applied for 10 min and the slides were counterstained with hematoxylin for 1 min. After staining, the slides were dehydrated in increasing alcohol concentrations, finishing with xylene, and mounted in glass slides in DPX solution (Merck Millipore, Massachusetts, US).

**Table 1:** Protocol for the immunohistochemical analysis of peritoneal samples.

Staining	Epitope retrieval buffer	Block	Antibody Diluent	Primary antibody
CD31	TRIS-EDTA Buffer (10 mM Tris Base, 1 mM EDTA, 0.05 % Tween 20, pH 9)	PBS block (5 min)	Antibody Diluent (ABCAM)	Recombinant Anti-CD31 antibody (Abcam Cat# ab182981) 1:1000 (1 h)
α-SMA	Citrate Buffer (10 mM Citric Acid, 0.05 % Tween 20, pH 6)	Peroxidase block (5 min)	Antibody Diluent (ABCAM)	Recombinant Anti-alpha smooth muscle actin antibody (Abcam Cat# ab265588) 1:1000 (1 h)
CD68	TRIS-EDTA Buffer	Peroxidase block (5 min)	Antibody Diluent (ABCAM)	Recombinant Anti-CD68 antibody (Abcam Cat# ab283654) 1:100 (1 h)
F4/80	Citrate Buffer	Peroxidase block (5 min)	Antibody Diluent (ABCAM)	Recombinant Anti-F4/80 antibody (Abcam Cat# ab300421) 1:5000 (1 h)

### IHC picture analysis

QuPath 0.4.4 software (University of Edinburgh, Edinburgh, UK) [16] was used to analyse the immunohistochemical images. A representative image was imported, and the “simple tissue detection” option used to mark the tissue. The “positive cell detection” option was used to quantify the positively stained cells. A script was generated and used for the batch analysis of the pictures (Appendix Script A1). Data was exported to an Excel spreadsheet and analysed using OriginPro 8 (OriginLab, Massachusetts, USA).

### Statistical analysis

Statistical analyses were conducted using OriginPro 8. Data are presented as means ± standard deviations. Statistical significance was determined using one-way ANOVA, Student's *t*-test, or two-way repeated measures ANOVA with a significance level of *p* < 0.05.

## Results

### Experimental setup

**Fig. 1:**
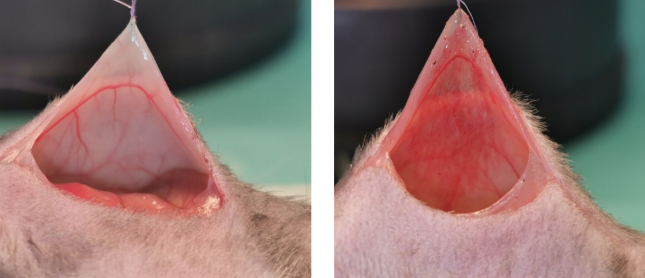
Abrasion examples for the mouse model. Animals were subjected to a 1 cm midline laparotomy. Left, peritoneum before abrasion. Right, erythematous reaction in the peritoneum following abrasion with a 100-grit Emery Cloth.

A representative example of the standardised abrasion to the peritoneum is shown in Fig. 1. Abrasion was performed until an obvious erythematous reaction was observed in the peritoneum.

### Post surgery Severity Assessment (PSSA) and pain levels

**Fig. 2:**
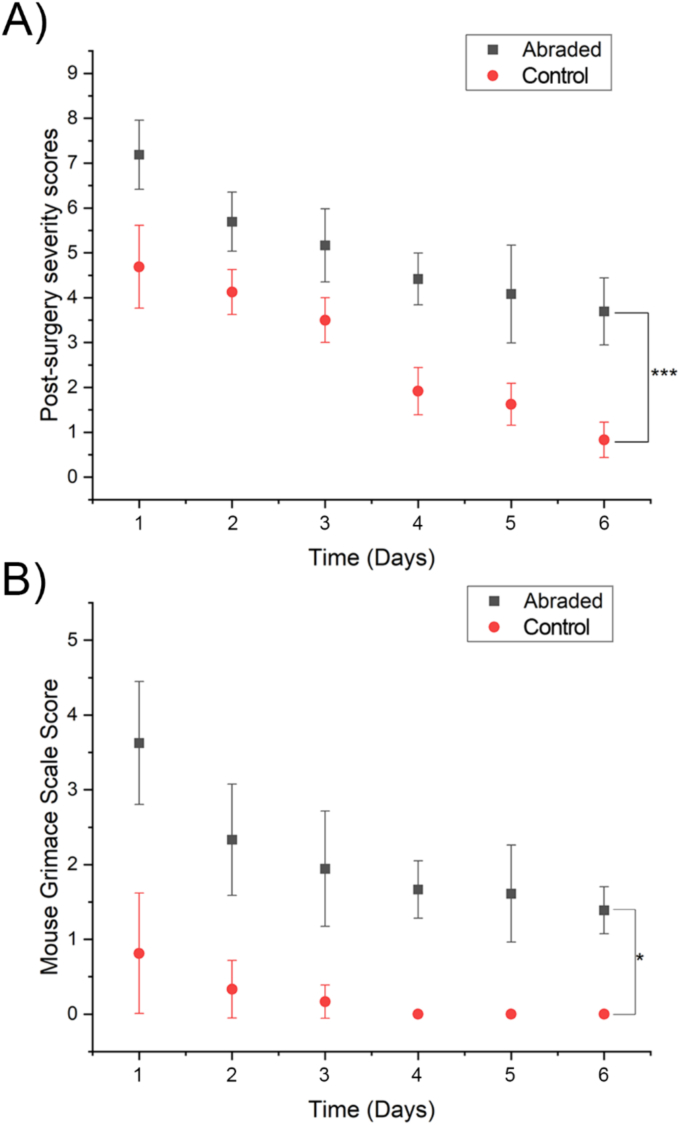
Post-surgery severity and mouse grimace scores after laparotomy. Comparison of Post surgery Severity Assessment scores (A) and Mouse Grimace Scale scores (B) observed in abraded (black) with unabraded mice (red) following recovery from the intervention. Two-way repeated measures ANOVA was used for statistical analysis, each data point represents the mean score with error bars (± sd). * represents a statistically significant difference, p ≤ 0.05. *** represents a statistically significant difference, p ≤ 0.001.

Both abraded and control animals showed an increased PSSA following laparotomy with a gradual reduction in the score across postoperative days 2 to 6 (Fig. 2A). The abraded group displayed significantly higher scores than the control group at each timepoint. At postoperative day 6, the abraded group still exhibited a low score, whereas the control group had returned to normal. A similar effect was observed for the Mouse Grimace Scale (Fig. 2B). The abraded group had a higher MGS on postoperative day 1 than the control group. By postoperative day 4, the MGS in the control group had returned to zero, while the abraded group still showed signs of pain even on the last day of the experiment an elevated MGS by postoperative day 6.

### Adhesion scores

**Fig. 3:**
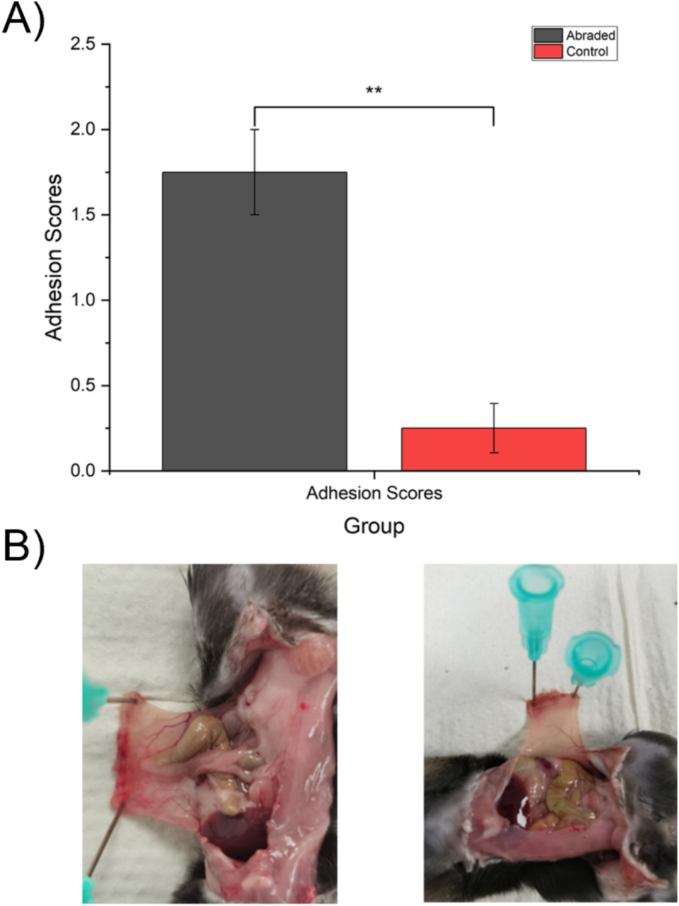
Adhesion scores results after schedule 1 of the animals. A) comparison of adhesion scores between the abraded and control mice groups. Each data point represents the mean adhesion score ± SD. ** represents a statistically significant difference using Student's t-test, p ≤ 0.01. B) Representative examples of adhesions in abraded group (left) and the lack of adhesion in the control group (right).

Following Schedule 1 killing, mice underwent laparotomy to determine whether adhesions were present. The adhesion scores of mice in the abrasion and control groups are shown in Fig. 3A. The abraded mice had a significantly higher occurrence of adhesions than the control group, where no adhesions were witnessed. A representative image from an abraded animal is shown in Fig. 3B.

### Cytokine quantification

**Fig. 4:**
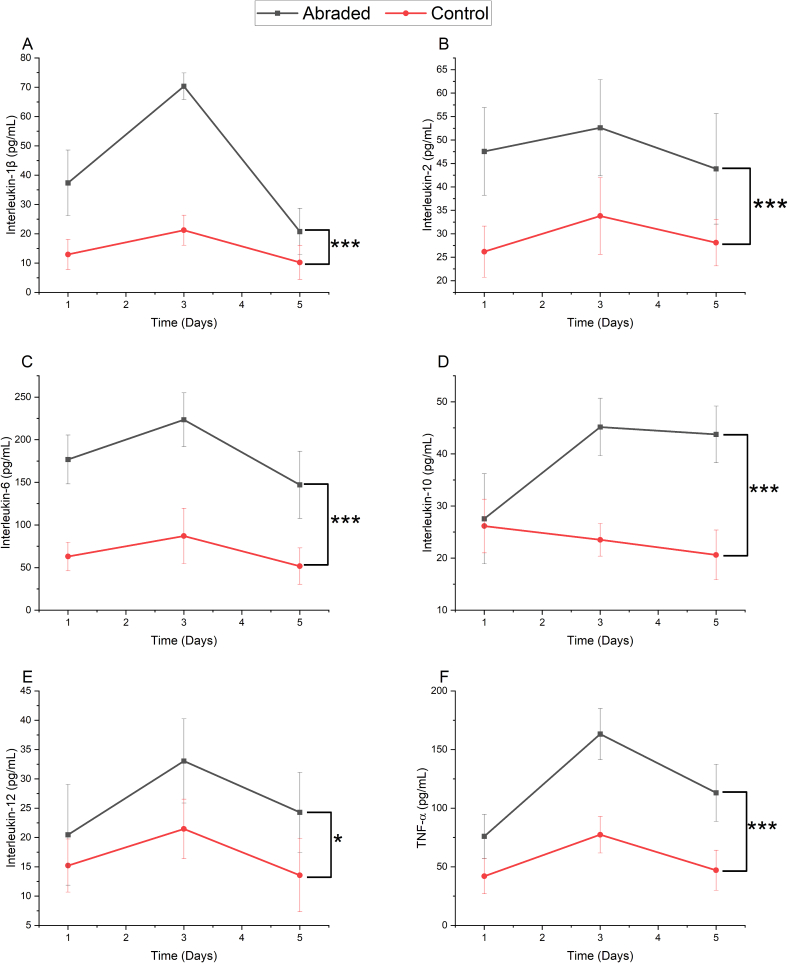
Cytokine quantification results from serum samples. Quantification (pg/mL) of A) IL-1β, B) IL-2, C) IL-6, D) IL-10, E) IL-12 and F) TNF-α from serum samples taken at days 1, 3, and 5 after surgery. The black line shows cytokine levels in the abraded group, and the red line in the control group. Two-way repeated measures ANOVA was used for statistical analysis, * represents a statistically significant difference, p ≤ 0.05. *** represents a statistically significant difference, p ≤ 0.001.

Fig. 4 shows the levels of serum cytokines IL-1β, IL-2, IL-6, IL-10, IL-12, and TNF-α in mice at days 1, 4, and 6 following surgery. Mice in the abrasion group showed higher cytokine levels than those in the control group at all three timepoints. There was a similar pattern of cytokine expression for all cytokines, with high levels on day 1, increasing by day 3, and decreasing by day 5.

### Immunohistochemistry

**Fig. 5:**
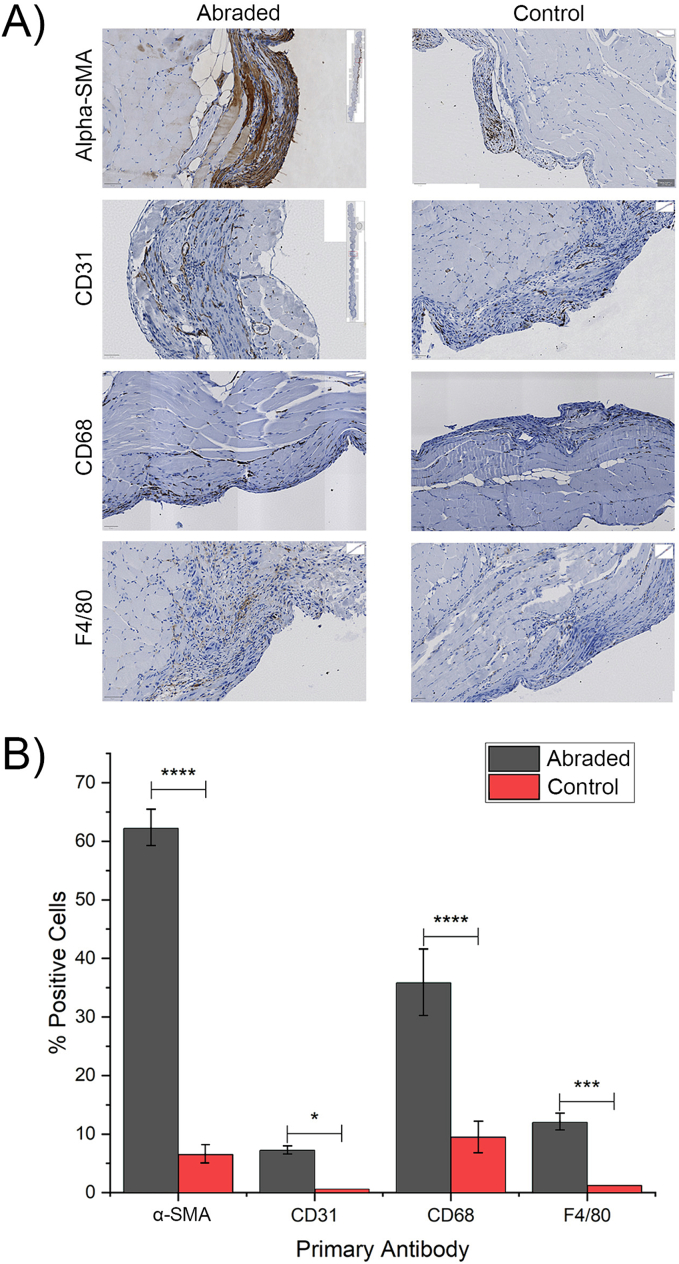
Immunohistochemistry results from peritoneal tissue samples. A) Percentage of positive cells expressing α-SMA, CD-31, CD68, and F4/80 from the abraded and control groups. Antibody expression was significantly higher in the abraded group. B) Examples of immunohistochemical staining for α-SMA, CD-31, CD68, and F4/80. Abraded samples showed greater immunoreactivity than control samples. ****, ***, * represents a statistically significant difference using Student's *t*-test p ≤ 0.0001, p ≤ 0.001, and p ≤ 0.05 respectively.

Representative images used for the analysis are shown in Fig. 5A. Fig. 5B shows the expression of α-SMA, CD31, CD68, and F4/80 in the abraded and control mice. The results show that the abraded group exhibited a statistically significant increase in expression of the antigens as compared to the control group. Notably, among the four antibodies tested, α-SMA and CD68 showed the highest difference in expression (*p* ≤ 0.0001), followed by F4/80 (*p* ≤ 0.001) and CD31 with the least significant difference (*p* ≤ 0.05).

## Discussion

Understanding the mechanisms that underlie postoperative pain is of great importance for clinical practice. It is fundamental to developing more sophisticated and safer pain-relieving strategies and, hence facilitating postoperative recovery. In this study, we have developed a mouse model that replicates acute pain following abdominal surgery. Our results show that by creating a standardised abrasion to the peritoneal lining of the abdomen, we can induce a painful response that lasts for the five days and is measurable using validated Post surgery Severity Assessment and Mouse Grimace Scores.

When assessing post surgical pain in mice, a multifactorial approach is necessary [17,18]. We have incorporated behavioural and physiological assessments into our model. Mice that had undergone peritoneal abrasion exhibited higher PSAA and MGS scores than the control group. Although the painful response for both groups decreased during the post operative observation period, the control group reached normal or near-normal scores around postoperative day 4, while abraded mice continued to show higher scores, reflecting the severity of the surgical injury.

An additional advantage of our model is the ability to evaluate adhesion formation; abraded mice showed significant adhesion formation, which was absent in control mice. The pathophysiology of adhesion formation is complex and has been studied using different animal models. Several cellular pathways are involved, some of which play a critical role in the normal process of wound healing [19]. The presence of adhesions in the abraded mice likely reflects down-regulation of peritoneal fibrinolytic activity and stimulation of a heightened inflammatory response [20].

We have further validated our model of postoperative pain by measuring inflammatory cytokine levels in the serum of mice at different time points following surgery. These inflammatory cytokines play a key role in the peritoneal response to trauma, initiating the inflammatory-immune response and playing an important role in peritoneal healing [21]. Among the cytokines measured, IL-6 is predominantly released from monocytes and corresponds to the severity of the injury [22]. The combined increased production of IL-6, IL-1β, and TNF-α leads to the recruitment of immune cells into the peritoneum and initiates collagen breakdown [23]. There is substantial evidence supporting the role of these cytokines in mediating pain; IL-1β [24], IL-6 [25], and TNF-α [26] are well-documented for their pro-inflammatory and pain-inducing effects, and there have been studies showing that these cytokines can sensitise nociceptive neurons, thereby contributing to pain [27]. Furthermore, the high levels of IL-6 compared to the other cytokines may be due to the fact that IL-6 also promotes the release of prostaglandins, which act as immunosuppressants to counteract inflammation [[21], [22], [23],^28^]. While the exact correlation between cytokine levels and pain perception may vary, the inflammatory response triggered by surgical trauma is likely to contribute significantly to the pain experienced.

The recruitment of proinflammatory cytokines leads to the recruitment of different types of inflammatory cells which infiltrate the peritoneum. Αlpha-SMA is produced in healing wounds by myofibroblasts and is an indicator of wound healing [29]. CD68 and F4/80 are both markers for macrophages which play a central role in phagocytosis and overall immune response regulation [30]. Our results indicate that increased peritoneal trauma caused by abrasion is associated by a proportionate increase in immune cells as part of the healing process. Despite both CD68 and F4/80 being markers for macrophages, CD68 is also expressed by monocytes (precursor cells to macrophages) [29]. CD31 is primarily expressed on endothelial cells and its expression is associated with angiogenesis which is critical for supplying nutrients and oxygen to healing tissue [31]. Our results show a higher CD31 expression in the abraded group, confirming the importance of angiogenesis to peritoneal healing.

## Conclusions

This study describes a novel model for assessing postoperative pain in mice. We have used a multi-dimensional approach to validate our model including assessment of behavioural, molecular, and cellular components of peritoneal inflammation and healing. We believe our model will be a valuable tool in facilitating the study of post surgical pain and for developing alternative strategies for pain relief.

## Funding sources

This work was supported by the University of Leeds' 10.13039/100010269Wellcome Trust institutional Translation Partnership Award (WT iTPA) [219420/Z/19/Z].

## Ethics approval

All experiments were performed according to the UK Animals (Scientific Procedures) Act (1986), The National Centre for the Replacement, Refinement and Reduction of Animals in Research (NC3Rs) Guidance and reported using International Animal Research: Reporting *In Vivo* Experiments (ARRIVE 2.0) Guidelines. Experiments were performed under UK Home Office Project Licence PP6019116.

## CRediT authorship contribution statement

**Juan Martinez:** Writing – original draft, Validation, Methodology, Investigation, Formal analysis, Conceptualization. **Thomas Maisey:** Validation, Resources, Methodology. **Nicola Ingram:** Writing – review & editing, Conceptualization. **Nikil Kapur:** Writing – review & editing. **Paul A. Beales:** Writing – review & editing, Supervision, Funding acquisition, Conceptualization. **David G. Jayne:** Writing – review & editing, Supervision, Conceptualization.

## Declaration of competing interest

None.

## Figures and Tables

**Appendix Table A1:** Post-surgery severity assessment score used for monitoring of the mice. Obtained from Zieglowski, Kümmecke [13].

Score	Body weight
0	Unaffected or increase
1	Decrease <5 %
5	Decrease <10 %
10	Decrease <19 %
20	Decrease ≥20 %


Score	General condition
0	Fur smooth, shiny; body orifices clean; eyes clear, shiny; unimpaired appearance
1	Minor fur defects, slight disordered fur, unkempt coat around eyes (red tears, eye ointment residues, etc.)
5	Dull or disordered, shaggy gur; unkempt body opening; eyes cloudy, slightly closed; slightly altered muscle tone (staggering writhing); frequent stretching (back arching, etc.)
10	Dirty fur, abnormal posture (raised back); high muscle tone; dehydration; altered breathing; diarrhoea (<48 h)
20	Cramps; paralysis; difficulty breathing; icterus; diarrhoea (<48 h); animal feels cold; permanent crouching with closed eyes


Score	Behaviour
0	Normal behaviour (sleeping, reaction to touch, curiosity, raising the edge of the cage, social contacts, social interaction, cleaning)
1	Minor deviations from normal behaviour; protective posture of the operated area during rearing
5	Unusual behaviour; motor abnormalities (stilt gait, protective posture of the operated area); reduced willingness to move (only after impulsion)
10	Isolation; apathy; strongly reduced willingness to walk; behavioural stereotypes/aggressiveness; coordination disorders
20	Pain noise during grabbing; lethargy; no readiness to mode; automutilation


Score	Surgery associated and trial specific parameters
0	Wound healing unimpaired, no swelling
1	Wound with minor redness, suture ends gnawed off (wound closed)
5	Wound redness, minor wound swelling, slight manipulation of the wound (wound closed)
10	Wound secretion, gnawing on wound, wound infection, suture dehiscence (discontinuous wound margins), minor postsurgical bleeding, swelling of tongue
20	Repeated suture dehiscence, severely infected wound, haemascosis, high grade swelling of the tongue with opened mouth, impaired breathing


Score	Assessment, actions
0	Assessment level 0: no severity, continue with regular monitoring
1–9	Assessment level 1: mild severity, increase monitoring (minimum twice a day) provide additional support (mashed food, medication)
10–19	Assessment level 2: moderate severity; increase monitoring (minimum 4 times per day), provide additional support (mashed food, medication, fluids, additional nesting), cull if no improvement in 24 h
>20	Assessment level 3: severe severity (humane endpoint); immediate termination of the experiment, euthanise animal

**Figure:**
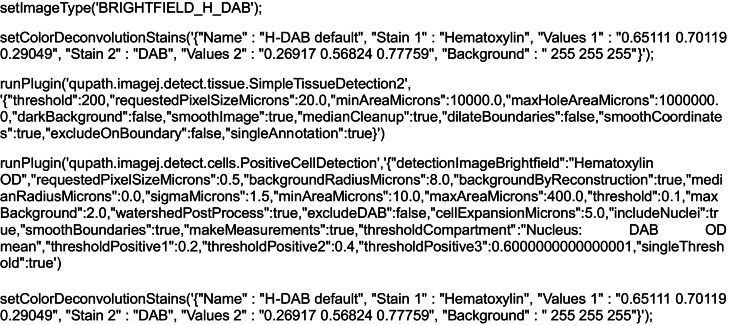


## Appendix A

### Appendix Script A1

The provided script serves as a batch processing tool designed for analysing immunohistochemical staining within the Qupath 0.4.4 software, developed by the University of Edinburgh, UK [16]. This script was automatically generated from the “command history” of Qupath during an image analysis session. To enhance its applicability, minor modifications were made to eliminate unnecessary steps.

It's important to note that this script may not be universally applicable to other projects due to potential variations in antibodies, staining conditions, and image acquisition methods. Nevertheless, it can be employed as a valuable template for conducting similar analyses.
